# Efficient Calibration of Groundwater Contaminant Transport Models Using Bayesian Optimization

**DOI:** 10.3390/toxics11050438

**Published:** 2023-05-06

**Authors:** Hao Deng, Shengfang Zhou, Yong He, Zeduo Lan, Yanhong Zou, Xiancheng Mao

**Affiliations:** 1 School of Geosciences and Info-Physics, Central South University, South Lushan Road, Changsha 410083, China; 2 Key Laboratory of Metallogenic Prediction of Nonferrous Metals and Geological Environment Monitoring, Central South University, Ministry of Education, Changsha 410083, China; 3 Key Laboratory of Non-Ferrous Resources and Geological Hazard Detection, Changsha 410083, China

**Keywords:** model calibration, groundwater contaminant transport, Bayesian optimization

## Abstract

Numerical modeling is a significant tool to understand the dynamic characteristics of contaminants transport in groundwater. The automatic calibration of highly parametrized and computationally intensive numerical models for the simulation of contaminant transport in the groundwater flow system is a challenging task. While existing methods use general optimization techniques to achieve automatic calibration, the large numbers of numerical model evaluations required in the calibration process lead to high computing overhead and limit the efficiency of model calibration. This paper presents a Bayesian optimization (BO) method for efficient calibration of numerical models of groundwater contaminant transport. A Bayes model is built to fully represent calibration criteria and derive the objective function for model calibration. The efficiency of model calibration is made possible by the probabilistic surrogate model and the expected improvement acquisition function in BO. The probabilistic surrogate model approximates the computationally expensive objective function with a closed-form expression that can be computed efficiently, while the expected improvement acquisition function proposes the most promising model parameters to improve the fitness to the calibration criteria and reduce the uncertainty of the surrogate model. These schemes allow us to find the optimized model parameters effectively by using a small number of numerical model evaluations. Two case studies for the calibration of the Cr(VI) transport model demonstrate that the BO method is effective and efficient in the inversion of hypothetical model parameters, the minimization of the objective function, and the adaptation of different model calibration criteria. Specifically, this promising performance is achieved within 200 numerical model evaluations, which substantially reduces the computing budget for model calibration.

## 1. Introduction

The contaminant transport in groundwater flow systems is a concealed, long-term, invertible process that has become a serious environmental problem and poses threats to sustainable development [1,2,3,4]. Understanding the dynamic characteristics of contaminant migration and retention is crucial for pollution assessment and contaminant remediation in soils, sites, and groundwater.

The numerical modeling techniques offer effective and robust tools to quantify, simulate, and predict the process of contaminants transport in groundwater flow systems. Over the past few decades, an enormous number of groundwater numerical modeling studies have been devoted to simulating the transportation and fate of contaminants [5,6,7,8,9,10,11,12,13]. The numerical modeling of contaminant transport is dependent on the dynamic governing equations, which include a series of model parameters that affect the model solutions, i.e., the simulation results. Thus, regardless of the dynamic governing equations and solution methods, numerical modeling is confronted with the non-uniqueness of simulation results, which limits the accuracy of simulation results. Aiming to generate accurate and meaningful simulation results, extensive efforts have been made in model calibration [2,14,15,16,17,18,19,20,21,22,23,24].

Initially, the model calibration is conducted manually. However, calibration of the model with only a few parameters would lead to a tedious and time-consuming “trial-and-error” process. Automatic model calibration, which is generally cast as an optimization problem, greatly alleviates the human workload to tune the model parameters and improves the efficiency of parameter calibration. To solve the highly nonlinear optimization problem derived from automatic model calibration, common optimization techniques such as gradient-based methods and global optimization methods are generally used. The gradient-based optimization is adopted in most model calibration approaches, such as those in the literature [25,26,27] and well-known software such as PEST [14], UCODE [28], and Dakota [29]. To find the minimum of objective functions, this category of methods involves multiple iterations to update the solution in terms of gradients. To estimate the gradient with respect to N model parameters,N+1(forward difference) or 2N+1 (center difference) times of numerical modeling are required. Moreover, gradient-based methods can lead to local minimum solutions, hindering the accuracy of simulation results. The other line of research uses global optimization methods. The exemplary methods include genetic algorithms [20,30], simulated annealing [31], adaptive cluster covering [16], shuffled complex evolution [32], differential evolution [33], particle swarm optimization [34], and ant colony optimization [35]. While these methods are shown to be effective in finding the global minimum of the objective function and improving the model accuracy, the significantly increased numbers of numerical model evaluations compared with the gradient-based method are the major concern. For a high-dimensional and long-term simulation, only a single evaluation of numerical modeling would lead to a high computational workload. However, common optimization techniques are not tailored to fit this characteristic and include multiple objective function evaluations, which lead to extremely expensive and even prohibitive computation for the model calibration scenario.

Over the past few decades, the surrogate model has been introduced to hydrological modeling. The surrogate model has been widely used in the probabilistic approach for groundwater model calibrations. The probabilistic approaches [36,37,38,39,40,41], e.g., the well-known MCMC-based DRAME algorithms [42,43], generally entail hundreds or thousands of numerical model evaluations and would lead to prohibitive computational overhead. The surrogate model is an approximation to the computationally intensive numerical model but can be evaluated in a much more efficient fashion. A series of data-driven surrogate models have been proposed, such as Bagging Multivariate Adaptive Regression Splines [44], neural networks [45], Gaussian processes [46], kriging [47], radial basis functions [48], support vector machines [49], and genetic programming [50]. While these surrogate models solidly reduce the computational cost, the generation of a surrogate model that well fits the numerical model still entails a large number of numerical model evaluations, and little effort has been paid to the tailoring of an efficient optimization algorithm for surrogate models.

This paper proposes a novel model calibration method based on the Bayesian optimization (BO) method. The BO method is a global optimization method to optimize objective functions that are time-consuming to estimate. The BO method has shown its success in parameter tuning in machine learning [51,52], materials design [53], and recommender systems [54]. Compared with common optimization techniques that find the minimum by extensively exploring the objective function, the BO method uses a surrogate model to approximate the objective function and ensures computational efficiency. In terms of the surrogate model, specifically, an acquisition function is devised for the minimization of the objective function and the avoidance of unnecessary objective function evaluations at certain model parameter spaces. This allows us to find the optimal objective function using a very small number of numerical modeling evaluations. The proposed method is applied to model the transport and retention of chromium in groundwater flow systems. The results demonstrate that the BO method is effective and efficient in calibrating numerical models of groundwater flow and contaminant transport.

## 2. Materials and Methods

**Figure 1:**
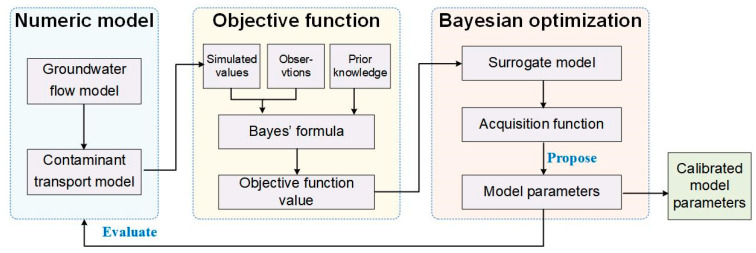
BO framework for calibration of the numeric model of groundwater contaminant transport.

Figure 1 illustrates the BO framework for calibration of the numeric model of groundwater contaminant transport. Given the numeric models of groundwater flow and contaminant transport, we first choose the target parameters to be calibrated. Then, an objective function is formulated to quantify calibration criteria. We use the Bayes’ rule to convert the calibration criteria into the objective function, in which we measure the fitness of simulated values to the observations and prior knowledge. To find the calibrated model parameters, the objective function is minimized by the BO method, in which the minimum of the objective function (i.e., optimized model parameters) is progressively searched in an iterated process. To reduce the expensive objective evaluations, the probabilistic surrogate model replaces the objective function in the BO method. In terms of the surrogate model, the acquisition function is defined to propose new model parameters for minimization of the objective function. Through specified numbers of BO iterations, the calibrated model parameters that well fit the calibration criteria can be obtained.

In the following, we will first derive the object function in Section 2.1 and then elaborate on the BO in Section 2.2, while the numeric models are introduced in Section 2.3.

### 2.1. Objective Function

Formally, we can represent the contaminant transport in the groundwater flow system as follows:

(1) $$\tilde{C}=M\left(X,\Theta \right)+\epsilon ,$$

where $\tilde{C}$ is the observation of contaminant concentration, $M\left(X,\Theta \right)$ is a numerical model that simulates the contaminant transport with input variables X and model parameters $\Theta ={\left\{\theta \right\}}_{i}^{n}$, and ϵ is the residual error. Here, the input X involves spatial-temporal locations of interest and boundary conditions. Given the set of observations $\tilde{C}={\left\{{\tilde{c}}_{i}\right\}}_{i=1}^{N}$, the posterior probability of model parameters Θ that guides model calibration can be formulated in terms of Bayes’ theorem:

(2) $$p\left(\Theta |\tilde{C}\right)=\frac{p\left(\tilde{C}|\Theta \right)p\left(\Theta \right)}{p\left(\tilde{C}\right)}\propto p\left(\tilde{C}|\Theta \right)p\left(\Theta \right),$$

where $p\left(\tilde{C}|\Theta \right)$ is the likelihood function that quantifies the fitness between simulation results and observations $\tilde{C}$, $p\left(\Theta \right)$ is the prior distribution $p\left(\Theta \right)$ that represents prior knowledge on model parameters $p\left(\Theta \right)$, and $p\left(\tilde{C}\right)=\int p\left(\tilde{C}|\Theta \right)p\left(\Theta \right)d\Theta$ is the evidence and normalizes $p\left(\Theta |\tilde{C}\right)$.

To calibrate the numerical model, we solve the model parameter Θ in a maximum a posteriori fashion, i.e., given the observations $\tilde{C}$, Θ is optimized such that its posterior probability $p\left(\Theta |\tilde{C}\right)$ is maximized:

(3) $$\Theta =\arg\underset{\Theta }{\max}p\left(\Theta |\tilde{C}\right)=\arg\underset{\Theta }{\max}p\left(\tilde{C}|\Theta \right)p\left(\Theta \right).$$

Here, by assuming that the observation error ϵ is normally distributed with zero means, the likelihood function can be defined in the form of Gaussian probability density:

(4) $$P\left(\tilde{C}|\Theta \right)=\prod_{i=1}^{N}\frac{1}{\sqrt{2\pi \sigma^{2}}}\exp\left(-\frac{{\left(M_{i}\left(X,\Theta \right)-{\tilde{c}}_{i}\right)}^{2}}{2\sigma_{i}^{2}}\right),$$

where $M_{i}\left(X,\Theta \right)$, ${\tilde{c}}_{i}$, and $\sigma_{i}$ are the numerical modeling result, the observation, and the standard deviation, respectively, at the i-th spatial-temporal location. Here, a standard deviation with a value of $\sigma_{i}$ indicates that it is roughly a 95% confidence that the simulation error $M_{i}\left(X,\Theta \right)-{\tilde{c}}_{i}$ falls into the interval of $\left(-2\sigma_{i},2\sigma_{i}\right)$ according to the 68–95–99.7 rule [55]. Thus, $\sigma_{i}$ is set according to a confidence level of the simulation error at i-th location and controls the fitness between $M_{i}\left(X,\Theta \right)$ and ${\tilde{c}}_{i}$.

On the other hand, we assume each parameter $\theta_{i}$ in the model parameters Θ is independent of the other parameters in Θ. Then, the joint prior probability $p\left(\Theta \right)$ of model parameters Θ is factorized as:

(5) $$p\left(\Theta \right)=\prod_{i=1}^{n}p\left(\theta_{i}\right).$$

For the prior probability $p\left(\theta_{i}\right)$, we can define it by using the kernel density estimation [56] if we have measurements on $\theta_{i}$. Otherwise, we define it as the uniform distribution over the interval $\left[\theta_{i}^{inf},\theta_{i}^{sup}\right]$: $\theta_{i}\sim U\left(\theta_{i}^{inf},\theta_{i}^{sup}\right)$, where $\theta_{i}^{inf}$ and $\theta_{i}^{sup}$ define the valid range of $\theta_{i}$.

Combining Equations (2)–(5) to ensure numeric stability and following the convention of optimization, we use the negative log-posterior as the objective function of model calibration:

(6) $$\underset{\Theta }{\min}f\left(\Theta \right)=\underset{\Theta }{\min}\left\{-\log p\left(\Theta |\tilde{C}\right)\right\}$$

### 2.2. Bayesian Optimization

The optimization problem in Equation (6) involves a “black-box” and computationally expensive objective function. In terms of such characteristics, the BO method is a promising approach to solving the optimization problem in Equation (6). The BO method uses a probabilistic surrogate model to represent the “black-box” objective function and sequentially refines (updates) the model with new sampling to the objective function. In contrast to gradient-based optimization, the BO method does not require knowledge about the gradients of the objective function and allows finding a global minimum for the non-convex objective function.

The key components of the BO method are the surrogate model and the acquisition model. The surrogate model is the probabilistic model of the objective function that represents our assumptions and knowledge of the “black-box” and the expected observations of the objective functions. The acquisition function evaluates a sampling for the objective function concerning the surrogate model, and the sequence of the samples for the objective function evaluation is generated by maximizing the acquisition function. Ideally, the minimum objective function can be found by optimizing a sequence of samples. For our scenario, each sample of the objective function leads to a simulation with the numerical model. The sampling updates the posterior probability of the surrogate model of the objective function. Algorithm 1 summarizes the workflow of BO for model calibration. Interested readers are referred to the literature [53,57,58,59] for more details of the BO method.
**Algorithm 1.** Bayesian optimization for model calibration.01 Initialize sample set $Q_{1}$ 02 for t=1,⋯,T−1 03   Update probabilistic surrogate model $p\left(f|Q_{t},\Theta \right)$ with $Q_{t}$ 04   Update acquisition function $\alpha \left(\Theta ;Q_{t}\right)$ with $p\left(f|Q_{t}\right)$ 05   Select the new model parameters $\Theta_{t}$ by maximizing the $\alpha \left(\Theta ;Q_{t}\right)$:              $\Theta_{t}\leftarrow {argmax}_{\Theta }\alpha \left(\Theta ;Q_{t}\right)$
06   Compute simulation results $M\left(X,\Theta_{t}\right)$ 07   Compute the objective function $f\left(\Theta_{t}\right)$ with $M\left(X,\Theta_{t}\right)$ 08   $Q_{t+1}=Q_{t}\cup \{\Theta_{t},f\left(\Theta_{t}\right)\}$ 09 end for 10 Output model parameters $\Theta^{*}$ with the smallest $f\left(\Theta \right)$


**Surrogate model.** Given the sampling results Q to the objective function, Gaussian process (GP) regression is generally used as the probabilistic surrogate model $p\left(f|Q,\Theta \right)$ for the computationally intensive objective function. The GP regression is flexible and scalable to model an arbitrary nonlinear function and requires a relatively small number of data examples. This is well-suited to model to objective function calibration. The GP extends the multivariate Gaussian distribution over vectors (finite-dimension space) to a distribution over functions (infinite-dimension space), for which any finite collection of dimensions follows a multivariate Gaussian distribution. The distribution of the Gaussian process is the joint distribution of infinite random variables. To surrogate the objective function $f\left(\Theta \right)$, the distribution of the Gaussian process for f is defined by a mean function $m\left(\Theta \right)$ and a covariance function $k\left(\Theta ,\Theta \prime \right)$:

(7) $$f\left(\Theta \right)\sim GP\left(m\left(\Theta \right),k\left(\Theta ,\Theta \prime \right)\right).$$

Following conventions [60], the prior mean function $m\left(\Theta \right)$ is assumed to be a mean function, while the covariance function is set to the squared exponential function. Then, given the known samples Q including t evaluations of objective function values: $f={\left(f\left(\Theta_{1}\right),\cdots ,f\left(\Theta_{t}\right)\right)}^{T}$, according to the Sherman–Morrison–Woodbury formula, a sample of objective function $f\left(\Theta \right)$ at Θ is derived to be Gaussian distributed [60]:

(8) $$P\left(f|Q,\Theta \right)=N\left(\mu \left(\Theta \right),\sigma^{2}\left(\Theta \right)\right),$$

where

(9) $$\mu \left(\Theta \right)=k^{T}K^{-1}f\;\sigma^{2}\left(\Theta \right)=k\left(\Theta ,\Theta \right)-k^{T}K^{-1}k,$$

$K\in R^{t\times t}$ is the covariance matrix of f, with the entry at the i-th row, the j-th column being $k\left(\Theta_{i},\Theta_{j}\right)$, and $k\in R^{t}$ is a vector with the i-th entry being $k\left(\Theta ,\Theta_{i}\right)$. In realistic cases, an evaluation of the objective function can include the residential error. Assuming the residential error is independent and identically Gaussian distributed with the zero mean and the variance $\sigma_{n}^{2}$, the covariance matrix of the noisy version of f becomes $K+\sigma_{n}^{2}I$, which can also avoid overfitting to the noise-corrupted objective functions [60]. Thus, the predictive surrogate model $p\left(f_{*}|Q,\Theta \right)$ is:

(10) $$P\left(f_{*}|Q,\Theta \right)=N\left(\mu_{*}\left(\Theta \right),\sigma_{*}^{2}\left(\Theta \right)\right),$$

where

(11) $$\mu_{*}\left(\Theta \right)=k^{T}{\left(K+\sigma_{n}^{2}I\right)}^{-1}f\;\sigma_{*}^{2}\left(\Theta \right)=k\left(\Theta ,\Theta \right)-k^{T}{\left(K+\sigma_{n}^{2}I\right)}^{-1}k.$$

Note that in Equation (10), the surrogate model with GP returns the posterior distribution of $f\left(\Theta \right)$ rather than its scalar value.

**Acquisition function.** Given the probabilistic surrogate model, as shown in Algorithm 1, the acquisition function is updated for optimization of model parameters and for determining the model parameters for the next sampling. The acquisition function considers the tradeoff between exploitation (i.e., sampling regions with small values of the objective function) and exploration (i.e., sampling regions with high uncertainty). The expected improvement (EI) criterion [61] is used as the acquisition function, which is indicated to be effective theoretically and empirically [62,63]. For our model calibration scenario, given known sampling results Q, the EI function α(Θ) is defined as the expected amount of improvement the new model parameters can yield:

(12) $$\alpha \left(\Theta \right)=E\left(\max\left\{0,f\left(\Theta^{+}\right)-f\left(\Theta \right)\right\}\right)=\int_{-\infty }^{+\infty }\max\left\{0,f\left(\Theta^{+}\right)-f\left(\Theta \right)\right\}p\left(f|Q,\Theta \right)df,$$

where $f\left(\Theta^{+}\right)$ denotes the smallest value of objective function given current sampling results Q. When $p\left(f|Q,\Theta \right)$ is defined as GP, the EI function has an analytic expression:

(13) $$\alpha \left(\Theta \right)=\left(\mu \left(\Theta \right)-f\left(\Theta^{+}\right)\right)\Phi \left(Z\right)+\sigma_{i}\left(\Theta \right)\phi \left(Z\right),$$

where $\Phi \left(\cdot \right)$ and $\phi \left(\cdot \right)$ are the cumulated distribution function and the distribution function of standard normal distribution, and

(14) $$Z=\frac{f\left(\Theta^{+}\right)-f\left(\Theta \right)}{\sigma_{i}\left(\Theta \right)}.$$

Unlike the objective function evaluation that includes numerical modeling, $\alpha \left(\Theta \right)$ only includes simple algebra calculations, such as $\mu \left(\Theta \right)$, $\Phi \left(Z\right)$, and $\phi \left(Z\right),$ and thus is inexpensive to estimate. Therefore, the gradient-based optimization can be used to maximize $\alpha \left(\Theta \right)$ in Algorithm 1, which can derive model parameters for the next sample efficiently.

### 2.3. Numerical Model

**Contaminant transport model.** The fate and transport of a contaminant (hexavalent chromium) in 3D transient groundwater flow systems are modeled by the Fickian advection-dispersion transport equation with retardation [64]:

(15) $$\vartheta R\frac{\partial c}{\partial t}=\nabla \cdot \left(\vartheta D\nabla c\right)-\nabla \cdot \left(qc\right)+q_{s}c_{s},$$

where c is the contaminant concentration (${ML}^{-3}$), t is the time ($T^{-1}$), R is the retardation factor, ϑ is the porosity, D is the dispersion coefficient tensor ($L^{2}T^{-1}$), q is the Darcy velocity ($LT^{-1}$), $q_{s}$ is the volume flow rate per unit volume of fluid sink/source ($T^{-1}$), $c_{s}$ is the contaminant concentration of fluid sink/source ($T^{-1}$).

The transport of contaminant through advection in porous media, represented by Darcy velocity q in Equation (15), is related to the groundwater flow equation by Darcy’s Law:

(16) q=−K∇h,

where K is hydraulic conductivity tensor ($LT^{-1}$) and h is the hydraulic head (L).

**Groundwater flow model.** The hydraulic head in the 3D groundwater flow system is modeled by the groundwater flow equation:

(17) $$S_{s}\frac{\partial h}{\partial t}=\nabla \cdot \left(K\nabla h\right)+q_{s},$$

where $S_{s}$ is the storage of aquifer ($T^{-1}$).

The finite difference method is adopted and discretizes and solves the above dynamic equation for groundwater flow and contaminant transport. In the finite difference method, the central difference scheme is used for spatial discretization, whereas the Crank-Nicolson scheme difference is used for time discretization. We developed a C++ program to implement the finite difference method, analogous to the implementations of MODFLOW [65] and MT3DMS [8].

## 3. Study Area

We applied the proposed model calibration method to simulate the transport of Cr(VI) in the groundwater flow system. Among the heavy metals, chromium, especially in its VI oxidation state, is extremely toxic and carcinogenic and has been added to the priority pollutant list by the United States Environmental Protection Agency (US EPA). The development of an accurate numerical model can provide in-depth insight into the migration and fate of Cr(VI) and guide post-remediation in the study area.

**Figure 2:**
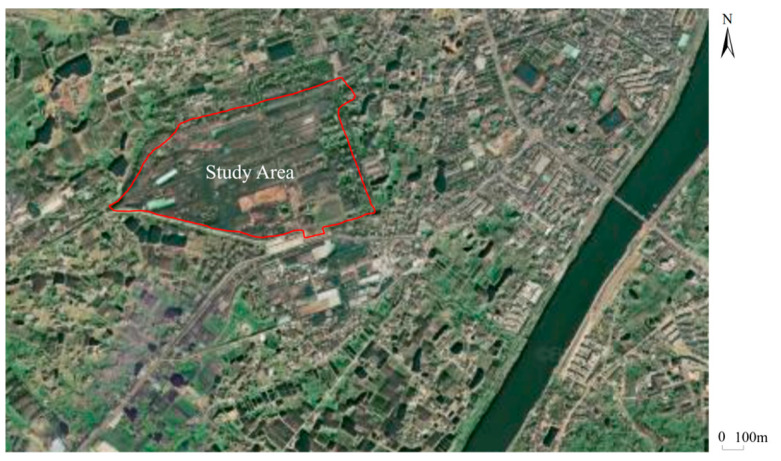
Arial image of the study area.

The study area is a Cr(VI)-contaminated site located in a city in Hunan province, south China (Figure 2). The region has a humid subtropical climate with an annual average temperature of 17.5 °C and an average annual precipitation of nearly 1400 mm. The site, with an area of 0.59 $km^{2}$ was originally an abandoned ferroalloy plant that produced 2000 tons of chromium metals annually. The production and processing of chromium lasted more than half a century at the site. It is estimated that overall 200,000 tons of chromium-bearing slags and tailings were produced during this period of history. Large amounts of chromium-bearing slags and tailings were directly dumped on the ground around the plant, leading to the leakage of chromium into the soil and groundwater.

The Cr(VI)-contaminated site is located on an alluvial stratum with fairly flat terrain. According to the engineering geology survey of the site, the alluvial stratum comprises four layers, including mixed fills, silty clays, sandy gravels, and weathered argillaceous siltstones from surface to depth. Table 1 summarizes the settings for the four layers. The layers of sandy gravels, which contain the confined pore water, are treated as a confined aquifer. The aquifer is characterized by weak permeability at its top and bottom boundaries, which can be regarded as an aquiclude due to the low vertical permeability. The major aquifer recharge comes mainly from the lateral flow of groundwater and rainfall. The recharge is considered to be uniform over the boundary, and lateral flow is roughly from northwest to southeast across the site.

**Figure 3:**
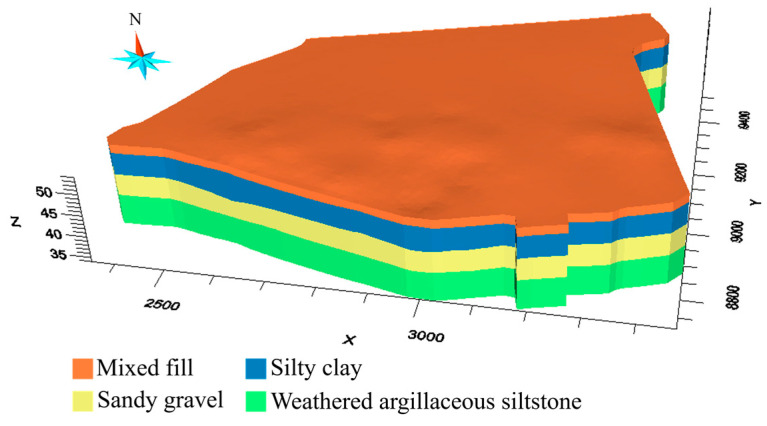
A 3D model of the study area.

In terms of the environmental survey, Cr(VI), Zn(II), Mn(II), Sn (III), Pb(II), Cd(II), and Na(I) have been detected in the groundwater. The concentration of Cr(VI) (maximum $mg\;L^{-1}$) significantly exceeds the referenced environmental limit (1.5 $mg\;L^{-1}$). Figure 3 shows the overserved Cr(VI) plume in 3D, which is generated by interpolation of the concertation of Cr(VI). The high concentration of Cr(VI) chromium (0.0005 $mg\;L^{-1}$~5.006 $mg\;L^{-1}$) is located in the central north of the site and extends from 5 m to 15 m beneath the surface.

## 4. Results

### 4.1. Setup

In our experiments, two cases are set up to investigate the effectiveness of the proposed method. The first one is a hypothetical case for testing the accuracy of model calibration and, especially, the identifiability of the hypothetical “true” values of model parameters. The second one is a real case for testing the fitness between simulated values and observations acquired in real fields.

**Figure 4:**
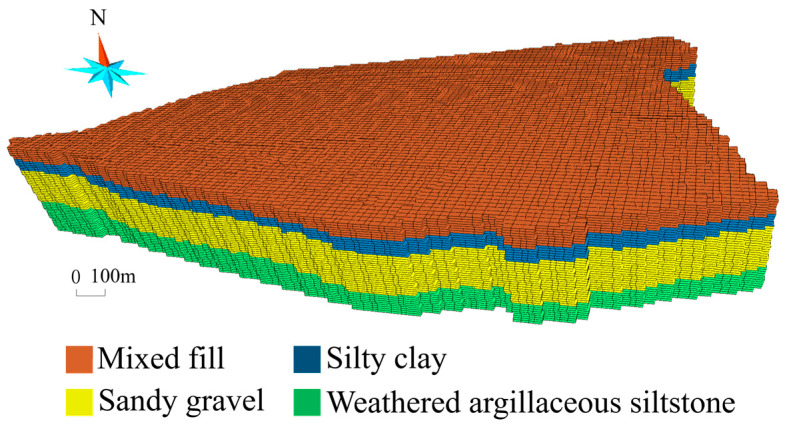
The 3D finite differential grids of the study area.

Before carrying out model calibration in the hypothetical and real cases, we reconstruct 3D models of the site (Figure 4) using drill-hole data at the site. The Cr(VI)-contaminated aquifer is discretized and modeled by a high-resolution finite-difference grid comprising 120×150×60=1,080,000 grids x, y, and z dimensions (Figure 4). Every grid is 10 m×10 m×0.5 m in size.

**Figure 5:**
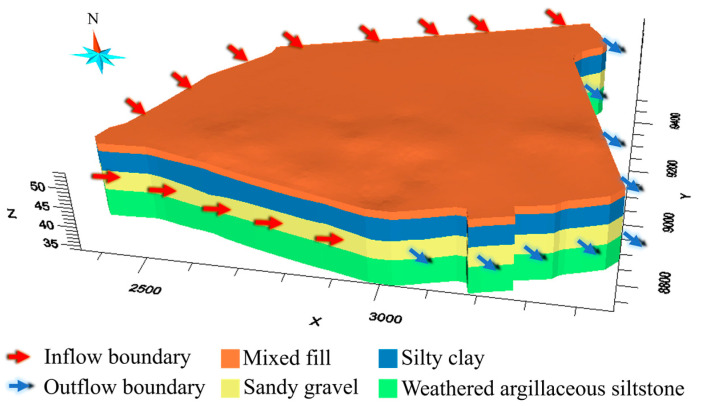
Boundary conditions for the groundwater flow model for the study area.

**Figure 6:**
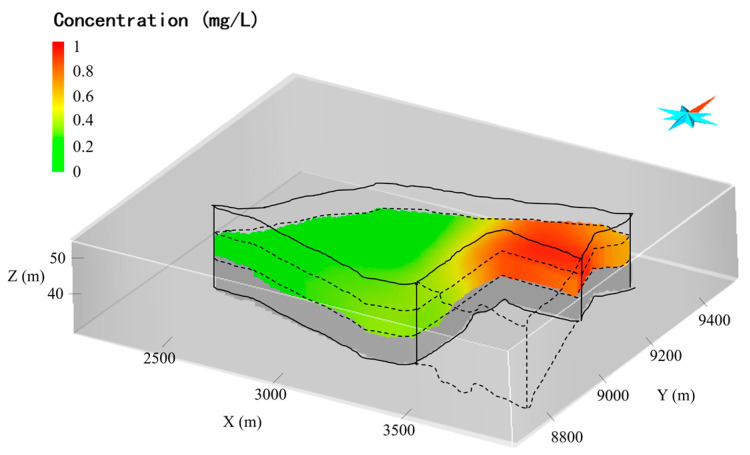
Initial concentration in the aquifer.

To simulate the groundwater flow, the boundary conditions for the flow model are shown in Figure 5. The northwest boundary condition specifies flux along the northwest boundaries, which represents the recharge of the lateral flow from the northwest of the site. The southeast boundary condition is a specified-head boundary that is set to a uniform constant. According to the hydrological survey for the site, a uniform recharge rate (3.0 $\times \;10^{-7}\;ms^{-1}$) is set for the northwest boundary, and 48 m is used as the water head for the outflow boundary (southeast boundary) according to the river stage in the vicinity of the site. The top boundary condition is also a flux-specific boundary condition to represent the recharge of rainfall at rates of $140\;cm\;{yr}^{-1}$ according to the annual precipitation of the region. The bottom boundary condition is a no-flow boundary since the argillaceous siltstones at the bottom of the aquifer can be considered impermeable. To simulate the transport of Cr (VI), the boundary conditions for the transport model are set to zero mass flux over all boundaries except the outflow boundary (i.e., the southeast boundary), where the mass flux is determined by the flow rate of the outflow through the boundary. The Cr(VI) concentration illustrated in Figure 6 is used as the initial condition for transport simulation.

We used the BO method to calibrate the parameters of groundwater flow and Cr(VI) transport models for the study area. Overall, six model parameters involved in Equations (15) and (17) were taken for model calibration, and their feasible ranges are listed in Table 2. We implemented the calibration method with Python, in which numerical simulations are called with a function implemented in C++ for the evaluation of objective functions. The BO method was run on an ordinary PC equipped with an Intel 3.6 GHz CPU, and only one computing thread was used.

In the following, we present the results of model calibration in the hypothetical case and the real case.

### 4.2. Hypothetical Case

**Figure 7:**
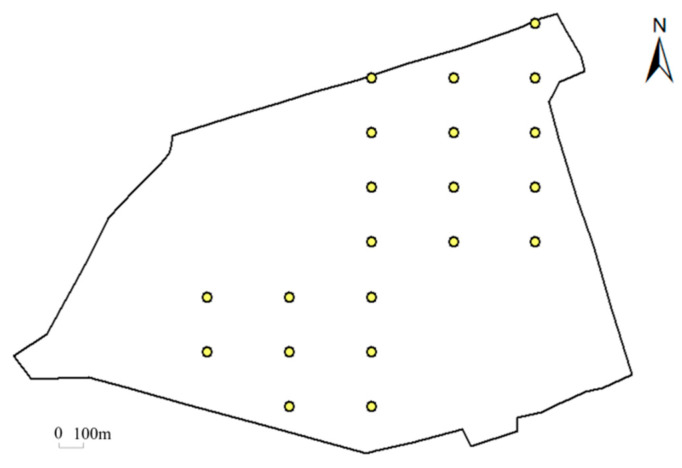
Spatial distribution of hypothetical monitoring wells.

Since real model parameters for the entire site are impossible to acquire, we set up a hypothetical case to validate the performance of the proposed method. Here, the “true” values of model parameters were specified beforehand, which are listed in Table 3. The “observations” were synthesized by the numerical model with the predefined model parameters (Table 3). To mimic the system stochastics and the measurement errors, Gaussian noises were added to the synthesized observations. The Gaussian noises are Gaussian distributed with a zero mean and the specified standard deviation (0.1 $mg\;L^{-1}$ was used if not stated otherwise). The synthesized data consists of observations at 21 monitoring wells (dots in Figure 7) in 32 weeks.

**Figure 8:**
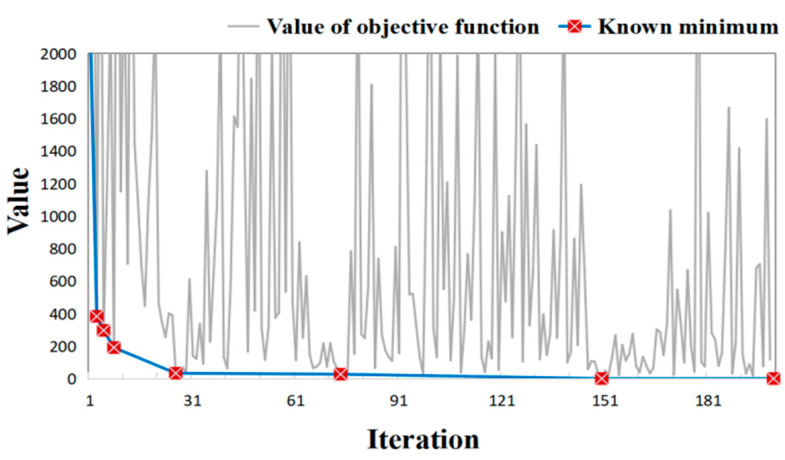
Variation of objective function values and the associated known minima during the BO process in hypothetical case.

Given the hypothetical observations, the BO method was used to optimize the objective function in Equation (3). Here, we set the maximum number of iterations to 200, which includes 200 times of numerical modeling. The overall model calibration process took 146 min. Figure 8 shows the variation of objective function values during the BO process. It is shown that the values of the objective function fluctuate significantly during iterations, reflecting that the BO process uses the acquisition function to probe the parameter space where the values of the objective function are relatively uncertain while exploiting the accumulated information to search for the minimum of the objective function. With the progression of BO iterations, the known minima of objective functions decrease substantially and gradually converge to zero (Table 4). The objective function considerably decreases after only 8 iterations (from an initial value >2000 to a value of 191.93). The BO process further searches for the minimum afterwards and finds the minimum (value = 1.03) at the 150th iteration.

To further investigate the model calibration accuracy, we compare the difference between simulated and observed concentrations. Especially, scatters of simulated concentrations versus observed concentrations, along with the 1:1 line and the associated 95% confidence interval lines, are visualized. To quantify the model calibration accuracy, the mean squared error (MAE) and the coefficient of determination ($R^{2}$) are used as the metrics, which are defined according to the notations in Equation (4):

(18) $$MAE=\frac{1}{N}\sum_{i=1}^{N}\left|M_{i}\left(X,\Theta \right)-{\tilde{c}}_{i}\right|,$$

(19) $$R^{2}=\frac{{\left(\sum_{i=1}^{N}\left(M_{i}\left(X,\Theta \right)-\overset{-}{M}\right)\left({\tilde{c}}_{i}-\overset{-}{c}\right)\right)}^{2}}{\sum_{i=1}^{N}{\left(M_{i}\left(X,\Theta \right)-\overset{-}{M}\right)}^{2}\sum_{i=1}^{N}{\left({\tilde{c}}_{i}-\overset{-}{c}\right)}^{2}},$$

where $\overset{-}{M}$ and $\overset{-}{c}$ are the mean of simulated concentrations and observed concentrations, respectively.

**Figure 9:**
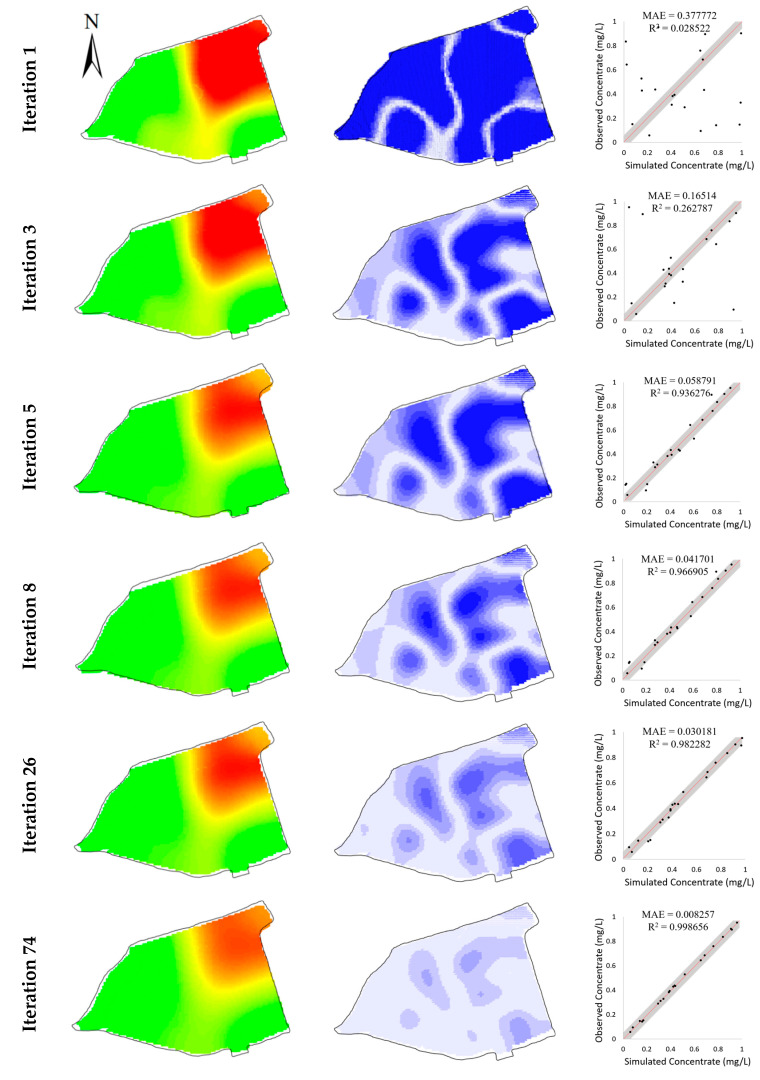

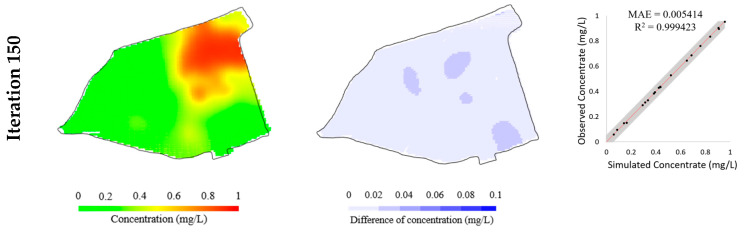
Evolution of simulated concentrations (**left column**), their differences from observed concentrations (**middle column**), and scatters of simulated versus observed concentrations (**right column**) during the BO process in hypothetical case.

Figure 8 illustrates the evolution of model performance during the BO iterations, in which the rows correspond to the iterations (except the first one) in Table 4 that find better solutions than the known minima. Analogous to the values of the objective function in Figure 9, the simulation errors are remarkably decreased, which demonstrates the ability of the BO to eliminate the simulation errors for the contaminant transport model.

**Figure 10:**
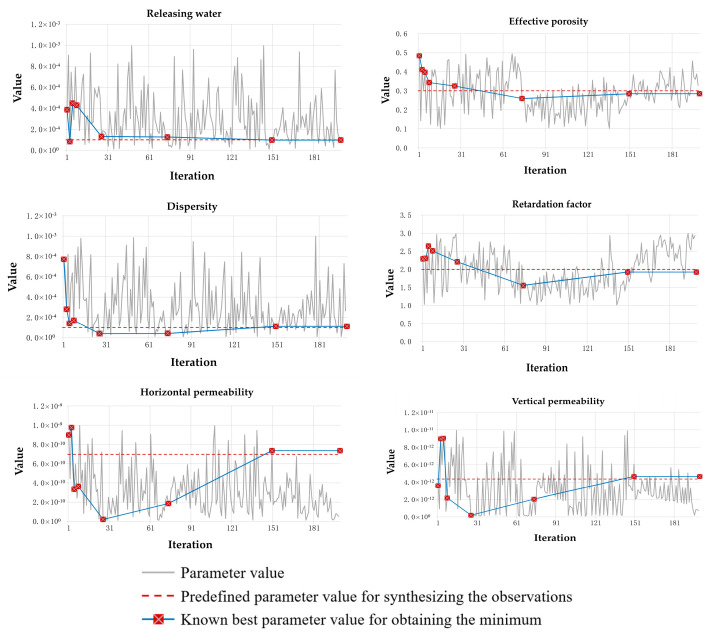
Variations model parameters during the BO process.

Figure 10 illustrates the variations of model parameters during the BO process, which further shows, by probing the objective function, how the model parameter space is explored. As observed in Figure 10, with the accumulated evaluations of objective functions, the values of parameters gradually approach their “true” values. This demonstrates that, given a limited number of objective function evaluations, the BO method can reasonably identify the model parameters for a relatively ideal contaminant transport system.

**Figure 11:**
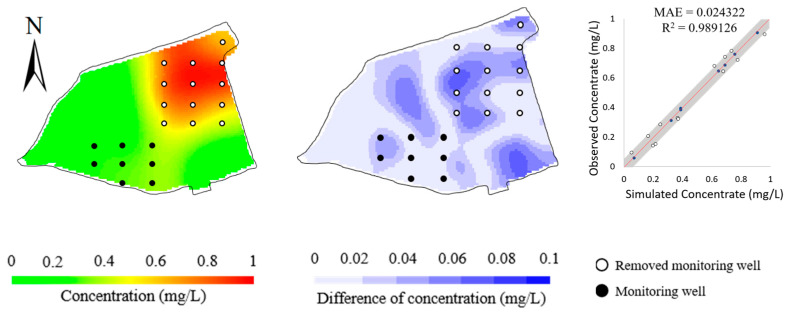
Simulated concentrations (**left column**), their differences from observed concentrations (**middle column**), and scatters of simulated versus observed concentrations (**right column**) obtained by removing the 13 monitoring wells in the north of the study area.

To validate the performance of the BO method in response to the observations, we designed an ablation study and a robustness study. In the ablation study, we removed the observations at the 13 monitoring wells in the north of the site and tested the impact on the model calibration. Figure 11 and Table 5 show that the simulation errors evidently increase for the removed 13 monitoring wells, whereas there is much less increase for the remaining 8 monitoring wells as calibration targets. This reflects that the BO method can be adaptive to the calibration targets. Table 5 summarizes the calibrated model parameters after removing the observations at the 13 monitoring wells. It is shown that the accuracy of the identified model parameters is slightly impacted, reflecting that the BO method is flexible to meet the set of observations. Note that the accuracy of horizontal and vertical permeability is evidently different. This may be attributed to the fact that the major transport mechanism of Cr(VI) in this study is advection, which is dominated by permeability. Given that the given concentrations at 8 monitoring wells in the south are much lower than the concentrations at 13 monitoring wells in the north, it is reasonable that the calibrated permeability is much deviated from its “true” values after removing the observations in the south. Thus, the calibrated model parameters suggest the ability of BO to explore the parameter space and fit the calibration targets.

**Figure 12:**
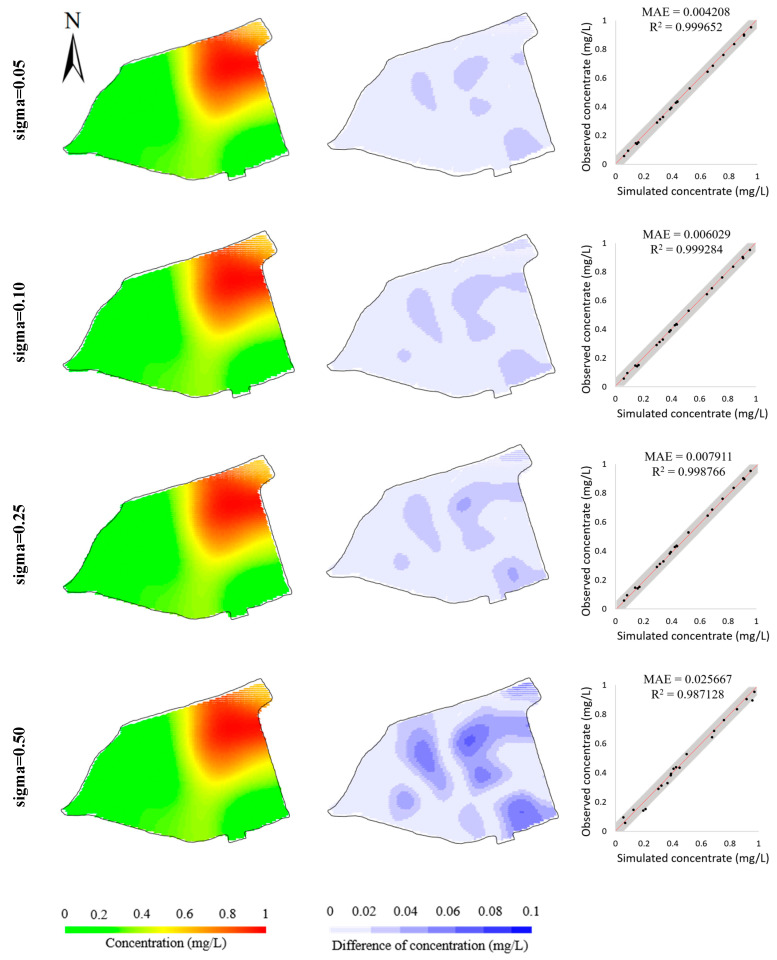
Comparisons of model calibration resulting from the calibration targets with different levels of Gaussian noise (Sigma/$mgL^{-1}$: standard deviation of the Gaussian noise added to calibration targets). Simulated concentrations (**left column**); differences from observed concentrations (**middle column**); scatters of simulated concentrations versus observed concentrations (**right column**) in hypothetical case.

The robustness study is designed to validate the robustness of the BO method to measurement error and other unknown system interference. Here, we enhanced the Gaussian noises added to the hypothetical observations. By using different standard deviations of Gaussian noise, four datasets of hypothetical observations were generated. Figure 12 illustrates the simulation errors of the four calibrated models. It is observed that, with the increase in standard deviation, the simulation errors increase slightly, demonstrating that the BO method is able to obtain an acceptable calibrated model even given the calibration targets generated from an unideal contaminant transport system. This is further verified by the inversed model parameters listed in Table 6, which show the inversed model parameters are kept close to the “true” model parameters.

**Figure 13:**
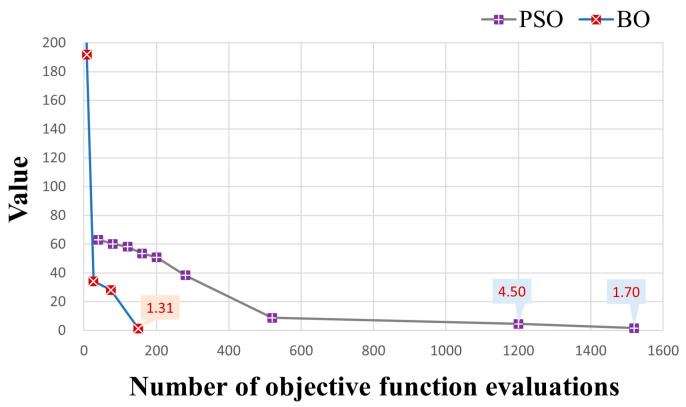
Comparison of objective function values and the associated known minima during the BO and the PSO iteration processes in hypothetical case.

**Figure 14:**
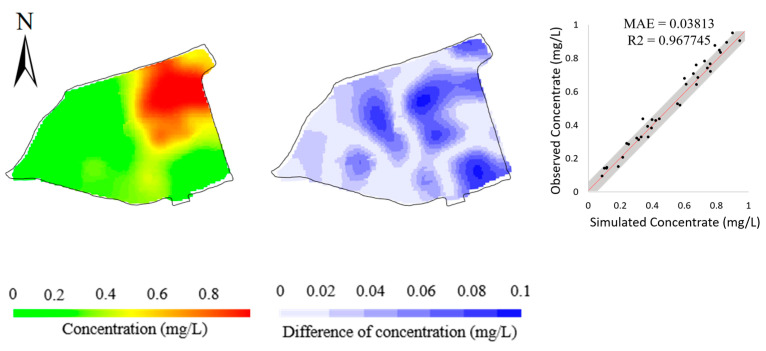
Simulated concentrations (**left column**), the differences from observed concentrations (**middle column**), and scatters of simulated versus observed concentrations (**right column**) resulting from the PSO method after 200 objective function evaluations.

To validate the efficiency of the BO method in model calibration, we compared it with Particle Swarm Optimization (PSO) [34], a well-known and effective model calibration method in the hydrological field due to its flexibility, ease of implementation, and efficiency [66,67]. The PSO method is a stochastic, population-based, global optimization technique inspired by swarm behavior observed in nature, such as fish and bird schooling. The number of particles is a significant hyperparameter in PSO. Following the setting in [34], an excessive number of particles (=40) was used for the efficiency of highly-parameterized model calibration. Here, an update of a particle’s position would lead to a numerical modeling process. To compare the efficiency between the PSO and BO methods, we stop the PSO iteration when the minimum of the objective function is close to or smaller than the minimum found by the BO method. Figure 13 illustrates the variations in objective function values during the PSO iterations and the BO iterations. It is noted that the PSO method took overall 1520 objective function evaluations (1109 min versus 146 min resulting from the BO method) to gain an objective function value (1.70) close to the minimum (1.31) of objective function found by the BO method, which is much more than 200 evaluations in the BO method. Figure 14 illustrates the model calibration performance of the PSO method after 200 objective function evaluations, which is the same number of objective function evaluations as that of the BO method. Compared with the results of the BO method (bottom row of Figure 9), the simulation error of the PSO method is significantly higher after the same number of objective function evaluations, which demonstrates the efficiency of the BO method in the calibration of Cr(VI) transport models.

### 4.3. Real Case

**Figure 15:**
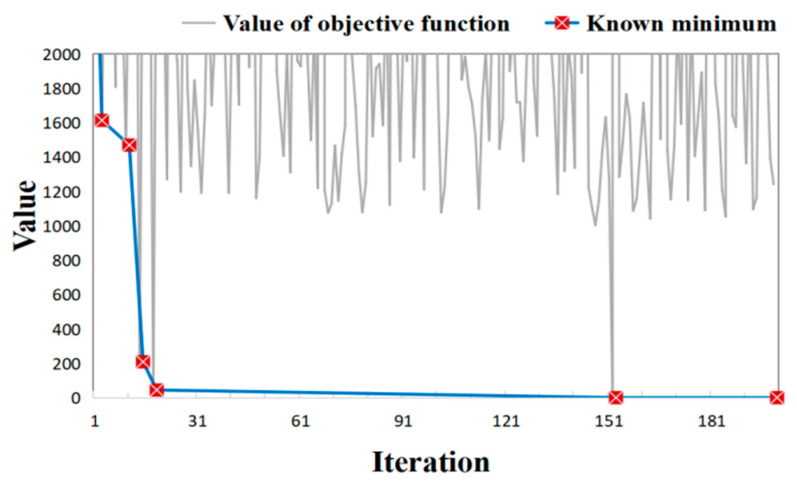
Objective function variation curve of the BO method process.

**Figure 16:**
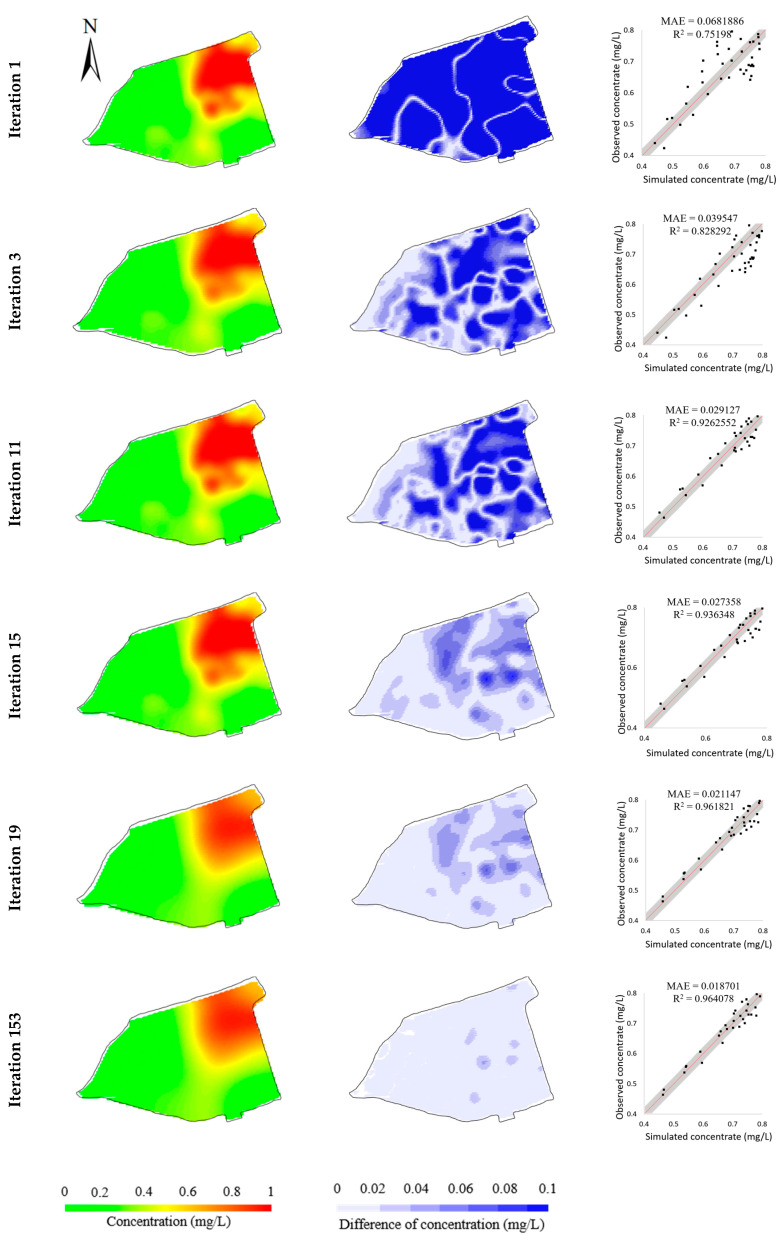
Evolution of simulated concentrations (**left column**), their differences from observed concentrations (**middle column**), and scatters of simulated versus observed concentrations (**right column**) during the BO process in real case.

While the BO method attains promising performance in the hypothetical cases, we further verify its effectiveness and reliability in a real case. These real data come from chromium slag sites in Hunan Province, China. Here, the Cr(VI) concentrations measured at 53 monitoring wells were set as the calibration targets. Since time series observations at these 53 bores are withheld by the stakeholder at the site, only the observations obtained seven months after the time the simulation starts are used for model calibration. The model calibration took 148 min. Figure 15 shows the variations of objective functions during the model calibration process, whereas Table 7 lists the values of objective functions at the iterations when a better solution is found. Analogous to the results in Figure 8, the values of the optimization function are minimized dramatically within two decades of iterations, which took only 15 min in the experiment. The minimum is found in the 153rd iteration, taking a computational time of 111 min. Figure 16 illustrates the evolution of simulated concentrations during the BO iterations, which shows that the simulation errors are considerably minimized during the BO process. Thus, the numerical model calibrated by the BO method is promising to fit the observations in the real-field scenario.

Figure 16 illustrates the evolution of simulated concentrations during the BO iterations, which shows that the simulation errors are gradually minimized during the optimization process. When the optimization reaches the minimum, the simulated concentration is within the 95% confidence interval, which indicates the calibrated model is promising to fit the observations.

**Figure 17:**
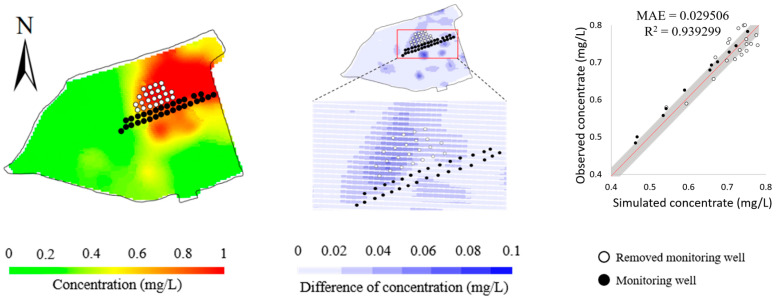
Simulated concentrations (**left column**), their differences from observed concentrations (**middle column**), and scatters of simulated versus observed concentrations (**right column**) obtained by removing the 23 monitoring wells in the north of the study area.

We further conducted the ablation study and the robustness study to verify the effectiveness and adaptivity of the BO. In the ablation study, we removed the observations at 23 monitoring wells in the north and only left the observations at 20 monitoring wells in the south as calibration targets. Figure 17 shows the model calibration performance. The simulation errors at the remaining 30 monitoring wells involved in model calibration are clearly smaller than those at the 23 removed monitoring wells. Combining the simulation error in Figure 16, it is indicated that the BO method is faithful to the input calibration targets and is capable of fitting the observations.

**Figure 18:**
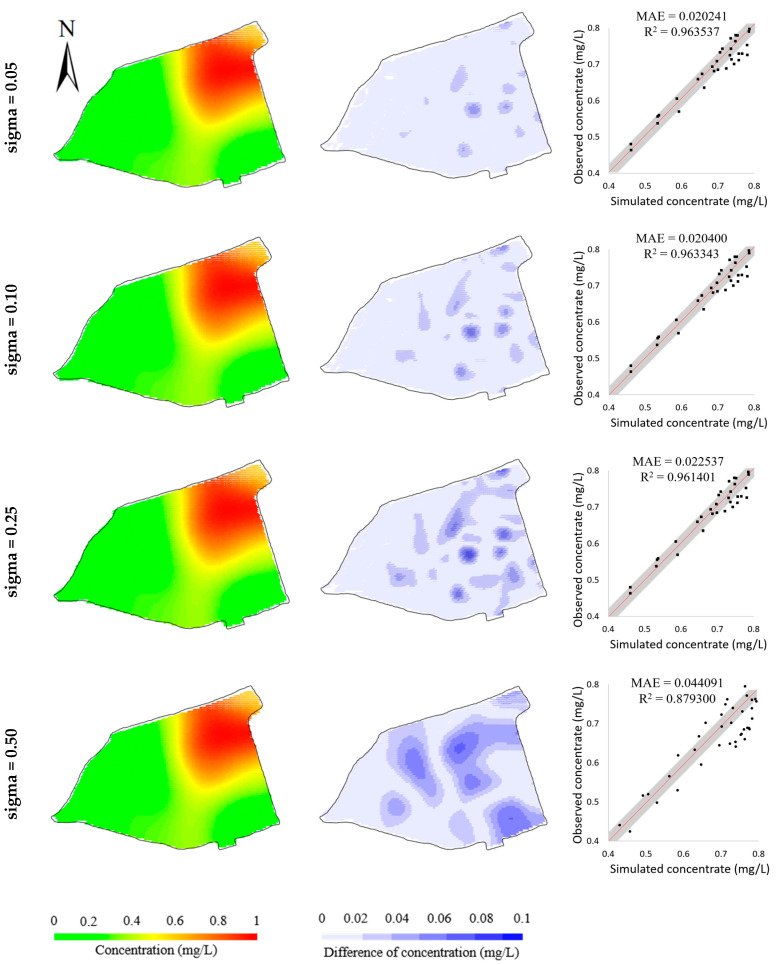
Comparisons of model calibration resulting from the calibration targets with different levels of Gaussian noise (Sigma/$mgL^{-1}$: standard deviation of the Gaussian noise added to calibration targets). Simulated concentrations (**left column**); differences from observed concentrations (**middle column**); scatters of simulated concentrations versus observed concentrations (**right column**) in real case.

In the robustness study, similarly to the hypothetical case, we added different levels of Gaussian noise to the observations. Overall, four standard deviations were used to generate the Gaussian noise. As shown in Figure 18, while the simulation errors are elevated due to the increase in Gaussian noises, it can be seen that the BO method is less sensitive to noises added to observations. Even though the noise added to the observations increases to 0.5, the MAE of model calibration remains approximately 0.03. This reflects that the BO method is robust enough to handle observation errors and possibly other zero-mean system errors in real-field applications. The observations errors can essentially affect the values of the objective function. In the BO method, the objective function is approximated by a surrogate model that explicitly considers noise-corrupted objective functions. Thus, it is reasonable that the model calibration results are robust to the noise of the objective function in practice.

**Figure 19:**
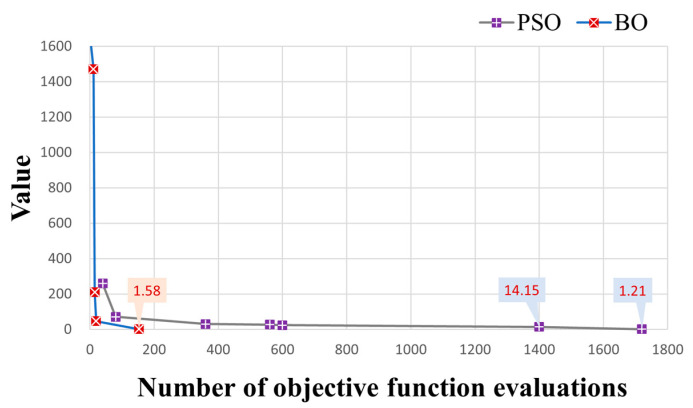
Comparison of objective function values and the associated known minima during the BO and the PSO iteration processes in real case.

**Figure 20:**
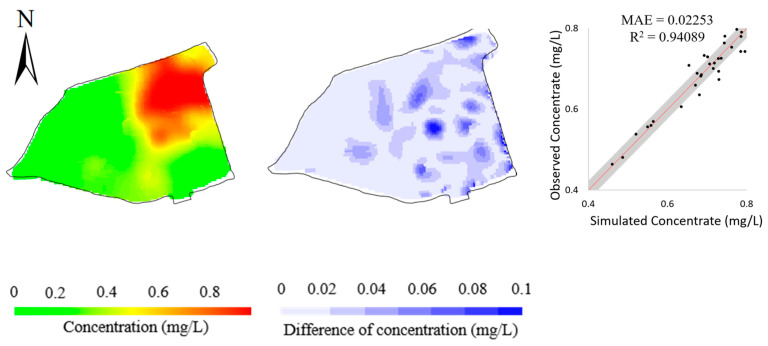
Simulated concentrations (**left column**), the differences from observed concentrations (**middle column**), and scatters of simulated versus observed concentrations (**right column**) resulting from PSO after 200 object function evaluations.

The BO method is further compared with the PSO method in the real case. Here, the setting of the PSO method is the same as that used in the hypothetical case. The PSO took 1720 object function evaluations (1255.6 min) to converge to an objective function value of 1.21 (Figure 19), the same order of magnitude as the objective function value (1.58) obtained by the BO method. When the number of numerical models is 200, the BO method evidently outperforms the PSO method (Figure 20). Combining the comparison results in both the hypothetic case and the real case, it is demonstrated that the BO method is evidently efficient in model calibration. In contrast to the stochastic PSO method, which is unaware of the structure of the objective function [68], the BO method uses the surrogate model to explicitly approximate the objective function and adopts an acquisition function to systematically search for the minimum objective function. Therefore, the BO method can efficiently search for the minimum of the objective function with fewer objective function evaluations.

## 5. Conclusions

In this paper, we propose a BO method for the calibration of 3D numerical models of groundwater flow and contaminant transport. A Bayes model is built to fully represent calibration criteria and derive the objective function for model calibration. In contrast to the use of common optimization approaches for model calibration, the computationally expensive objective function is approximated by a computationally efficient probabilistic surrogate model in BO. An effective acquisition function is adopted to search for the most promising model parameters that facilitate minimizing the objective function while exploring the highly uncertain region of the objective function. This enables finding the optimized model parameters effectively and efficiently by minimizing the objective function with a small number of objective function evaluations.

Two case studies of model calibration for Cr(VI) transport simulation, which involve the *hypothetical case* and the *real case*, demonstrate that, as the BO process proceeds, the BO method is effective in identifying hypothetical model parameters, minimizing the objective function, and adapting different calibration criteria. The promising performance of the BO method is achieved within 200 objective function evaluations, which took 111 min as reported on an ordinary PC. Compared with the PSO model calibration method, the BO method shows superior efficiency in both the *hypothetical* and *real case*.

In the future, we will further validate the performance of the BO method in more challenging scenarios and expand its applicability and flexibility. The performance of the BO method motivates the use of high-resolution numerical models for long-term and high-fidelity simulation. Due to the evidently lower number of objective function evaluations in BO, the high-resolution numerical simulations that were originally computationally prohibitive in the model calibration can now be allowed in applications. Another avenue is to scale the BO method to numerical models with hundreds of model parameters. This would allow us to comprehensively consider the spatial heterogeneity of model parameters in the numerical models. These potential improvements can further enforce the fitness of observations and result in more convincing numerical models for the simulation of contaminant transport in the groundwater flow system.

## Figures and Tables

**Table 1:** Characteristics of soil layers in the study area.

Layer	Thickness	Water Permeability
Mixed fill	3~4 m	Strong permeability
Silty clay	2~4 m	Weak permeability
Sandy gravel	3~4 m	Medium permeability
Weathered argillaceous siltstone	9~10 m	Weak permeability

**Table 2:** Model parameters.

Model Parameter	Value
Releasing water (m^−1^)	0.0$0\times 10^{-3}$~$1.00\times 10^{-3}$
Effective porosity	0.10~0.60
Dispersity (m^2^ s^−1^)	0.0$0\times 10^{-3}$~$1.00\times 10^{-3}$
Retardation factor	1.00~5.00
Horizontal permeability (m s^−1^)	$6.90\times 10^{-5}$~$8.30\times 10^{-5}$
Vertical permeability (m s^−1^)	$6.90\times 10^{-7}$~$8.30\times 10^{-7}$

**Table 3:** Target parameters.

Model Parameter	Value
Releasing water (m^−1^)	1.0 $0\times \;10^{-4}$
Effective porosity	0.30
Dispersity (m^2^ s^−1^)	1.00 $\times \;10^{-4}$
Retardation factor	2.00
Horizontal permeability (m s^−1^)	6.97 $\times \;10^{-10}$
Vertical permeability (m s^−1^)	4.37 $\times \;10^{-12}$

**Table 4:** Statistics of iterations and minima when a better solution of the objective function minimum is found.

Iteration	1	3	5	8	26	74	150
Minimum	2284.01	383.84	297.55	191.93	34.18	28.09	1.31

**Table 5:** Comparison of the model parameters identified with partial data and the full data shown in Figure 7. The partial data, compared with the full data, only include observations at eight monitoring wells in the south.

Model Parameter	Partial Data	Full Data	Target Parameters
Releasing water (m^−1^)	9.00 $\times \;10^{-5}$	1.00 $\times \;10^{-4}$	1.0 $0\times \;10^{-4}$
Effective porosity	0.31	0.28	0.30
Dispersity (m^2^ s^−1^)	1.10 $\times \;10^{-4}$	1.10 $\times \;10^{-4}$	1.00 $\times \;10^{-4}$
Retardation factor	1.90	1.92	2.00
Horizontal permeability (m s^−1^)	3.58 $\times \;10^{-10}$	7.35 $\times \;10^{-10}$	6.97 $\times \;10^{-10}$
Vertical permeability (m s^−1^)	1.82 $\times \;10^{-12}$	6.07 $\times \;10^{-12}$	4.37 $\times \;10^{-12}$
Objective function value	9.87	1.31	-

**Table 6:** Calibrated model parameters resulting from calibration targets with different levels of noise (sigma/$mgL^{-1}$ : standard deviation of the Gaussian noise added to calibration targets).

**Model Parameter**	Sigma = 0.05	Sigma = 0.10	Sigma = 0.25	Sigma = 0.50	Target Parameters
Releasing water (m^−1^)	1.10 $\times \;10^{-4}$	1.0 $0\times \;10^{-4}$	1.10 $\times \;10^{-4}$	1.1 $0\times \;10^{-4}$	1.0 $0\times \;10^{-4}$
Effective porosity	0.30	0.31	0.30	0.31	0.30
Dispersity (m^2^ s^−1^)	1.00 $\times \;10^{-4}$	1.20 $\times \;10^{-4}$	1.0 $0\times \;10^{-4}$	1.00 $\times \;10^{-4}$	1.00 $\times \;10^{-4}$
Retardation factor	1.96	1.98	1.95	2.08	2.00
Horizontal permeability (m s^−1^)	5.71 $\times \;10^{-10}$	5.88 $\times \;10^{-10}$	7.84 $\times \;10^{-10}$	6.02 $\times \;10^{-10}$	6.97 $\times \;10^{-10}$
Vertical permeability (m s^−1^)	4.80 $\times \;10^{-12}$	6.06 $\times \;10^{-12}$	3.98 $\times \;10^{-12}$	4.87 $\times \;10^{-12}$	4.37 $\times \;10^{-12}$
Objective function value	1.79	2.64	1.63	1.92	-

**Table 7:** Descending process of the optimal objective function.

Iteration	1	3	11	15	19	153
Known minimum	2975.10	1614.30	1470.65	210.01	46.03	1.58

## Data Availability

Not applicable.
